# Electrospinning of Nanodiamond-Modified Polysaccharide Nanofibers with Physico-Mechanical Properties Close to Natural Skins

**DOI:** 10.3390/md14070128

**Published:** 2016-07-07

**Authors:** Mina Mahdavi, Nafiseh Mahmoudi, Farzad Rezaie Anaran, Abdolreza Simchi

**Affiliations:** 1 Department of Materials Science and Engineering, Sharif University of Technology, Azadi Avenue, 14588 Tehran, Iran; mina_m1372@yahoo.com (M.M.); Nafiseh_Mahmoudi@mehr.sharif.ir (N.M.); fd_rezaieanaran@dena.sharif.ir (F.R.A.); 2 Institute for Nanoscience and Nanotechnology, Sharif University of Technology, P.O. Box 11365-9466, Azadi Avenue, 14588 Tehran, Iran

**Keywords:** nanodiamond, chitosan nanofiber, bacterial cellulose, electrospinning, wound dressing

## Abstract

Electrospinning of biopolymers has gained significant interest for the fabrication of fibrous mats for potential applications in tissue engineering, particularly for wound dressing and skin regeneration. In this study, for the first time, we report successful electrospinning of chitosan-based biopolymers containing bacterial cellulous (33 wt %) and medical grade nanodiamonds (MND) (3 nm; up to 3 wt %). Morphological studies by scanning electron microscopy showed that long and uniform fibers with controllable diameters from 80 to 170 nm were prepared. Introducing diamond nanoparticles facilitated the electrospinning process with a decrease in the size of fibers. Fourier transform infrared spectroscopy determined hydrogen bonding between the polymeric matrix and functional groups of MND. It was also found that beyond 1 wt % MND, percolation networks of nanoparticles were formed which affected the properties of the nanofibrous mats. Uniaxial tensile testing of the woven mats determined significant enhancement of the strength (from 13 MPa to 25 MP) by dispersion of 1 wt % MND. The hydrophilicity of the mats was also remarkably improved, which was favorable for cell attachment. The water vapor permeability was tailorable in the range of 342 to 423 µg·Pa^−1^·s^−1^·m^−1^. The nanodiamond-modified mats are potentially suitable for wound healing applications.

## 1. Introduction

Structural disruption in basement membranes of skin due to different types of injuries have to be cared and repaired as the skin is the largest organ of the body with many vital functions [1]. Elastic, biocompatible, and non-allergenic wound dressing materials are commonly utilized with an aim to keep the injured environment moist while protecting it from new infections [2]. With the same morphology and structure as the natural extracellular matrix (ECM), polymeric fibers are the most suitable materials that can be utilized for skin regeneration [3]. The porosity, oxygen permeability, mechanical durability, and hydrophilicity of fibrous membranes can be adjusted to practical needs by chemical and structural modifications [4].

So far, chitosan (CS) and CS-based fibers have been the most studied natural polymeric materials for wound healing and skin tissue engineering applications [5]. Not surprisingly, this natural polysaccharide offers a number of advantages over other natural and synthetic polymers. As a derivate of chitin [6], CS has proved to be biocompatible with non-allergenic activity and bactericidal capacity [7,8,9]. Perhaps the main drawback of CS is in regard to its relatively low mechanical durability, low structural stability in a physiological environment, and its degradation products [10]. From a processing point of view, high viscosity of CS solution may insert some restrictions on the fabrication of long and uniform fibers with controllable sizes [11]. Although electrospinning is one of the most versatile techniques to fabricate fibrous mats of various biopolymers in a cost-effective way [12], viscous CS solution is hard to be electrospun without utilizing co-surfactants and organic solvents (which may not be biocompatible) [13]. Recent studies have shown that CS fibers can be electrospun through blending with synthetic polymers; examples include PVA, PEO, PCL, and PVP [14,15]. The synthetic polymers reduce the viscosity of CS solution making it spinnable while improving the mechanical durability of fibrous mats. Recent advances are focused on utilizing natural polymers in order to tailor the properties of CS fibers. Bacterial cellulose (BC), which is produced by the bacterium *Acetobacter xylinum*, is a good candidate. This is because the ultrafine network of uniaxial cellulose nanofibers (3–8 nm) [16] terminates as a large surface area that can hold a large amount of water (up to 200 times of its dry mass) while providing a great elasticity, wet mechanical durability, and conformability [17]. The hydrophilicity [18], biocompatibility [19], and non-toxicity [20] of BC have also made this natural polymer a promising candidate for many biomedical applications [21,22]. Transparency and flexibility of CS/BC films prepared by solvent casting methods were studied by Fernandes et al. [23]. Phisalaphong et al. [24] added CS to the culture medium of BC during biosynthesis to attain CS/BC blends. Enhanced mechanical properties and water absorption capacity of the composite blend was shown. Additionally, the blends did not exhibit an adverse effect on in vitro cell viability [25]. The fabrication of CS/BC nanofibers has recently been attempted [26]. Very recently, Azarnia et al. [27] utilized the surface methodology approach to determine a set of optimum parameters to attain long, uniform, and fine CS/BC fibers. They showed how electrospinning parameters affected the morphology and size of the fibers and examined the physico-mechanical properties of the woven mats.

Although CS-based fibrous mats are promising candidates for wound dressing, the mechanical properties of the membranes do not meet the requirements of natural skin. In order to improve the mechanical durability and to tailor physical properties (permeability and hydrophilicity) of the electrospun membranes, the addition of secondary (reinforcing) particles to the polymer network has been studied [28,29]. Particular attention has been paid to carbon nanostructures [27,30,31]. Graphene-modified nanofibers have gained much interest in recent years [32]. It has been shown that graphene oxide and reduced graphene oxide nanosheets can be embedded in or folded around the polymer fibers to boost the mechanical strength of biopolymers, while providing bactericidal capacity along with enhanced cell attachment [27,33]. In the present work, biomedical grade nanodiamonds (MND) were utilized for the modification of CS/BC fibers. To the best knowledge of the authors, MND has not been utilized in wound dressing applications so far, despite the high potential of the material for biomedical applications. Nanodiamonds have been shown to be biocompatible in various cell lines and some animal models with minimal or no cytotoxicity [34,35]. The particular feature of MND that distinguishes them from other carbon nanostructures is related to their ultrafine sizes with many surface functional groups that have made them excellent carriers for drug delivery [36]. The functional groups provide stable colloids in aqueous solutions with an ability to conjugate to various drugs and growth factors. Therefore, the main objective of this work is to introduce MND to biopolymers in order to: (a) ease electrospinning of concentrated CS solutions; (b) decrease fiber diameters to nano-scale range; (c) enhance the mechanical durability of the membranes to be closer to that of the natural skin in the back area; and (d) provide a platform to conjugate drugs and growth factors to the nanoparticles for controlled release. In this first stage, and within this paper, we present experimental results on the electrospinning of MND-modified CS/BC nanofibers. The effects of MND on the spinnability, mechanical properties, hydrophilicity, water vapor permeability, and cytocompatibility are also shown. It is worthwhile to mention that this work is particularly focused on materials processing and characterizations, but further studies are underway to investigate the drug-loading ability of the MND-modified nanofibers structure.

## 2. Results and Discussion

### 2.1. Size and Morphology of Electrospun Fibers

**Figure 1:**
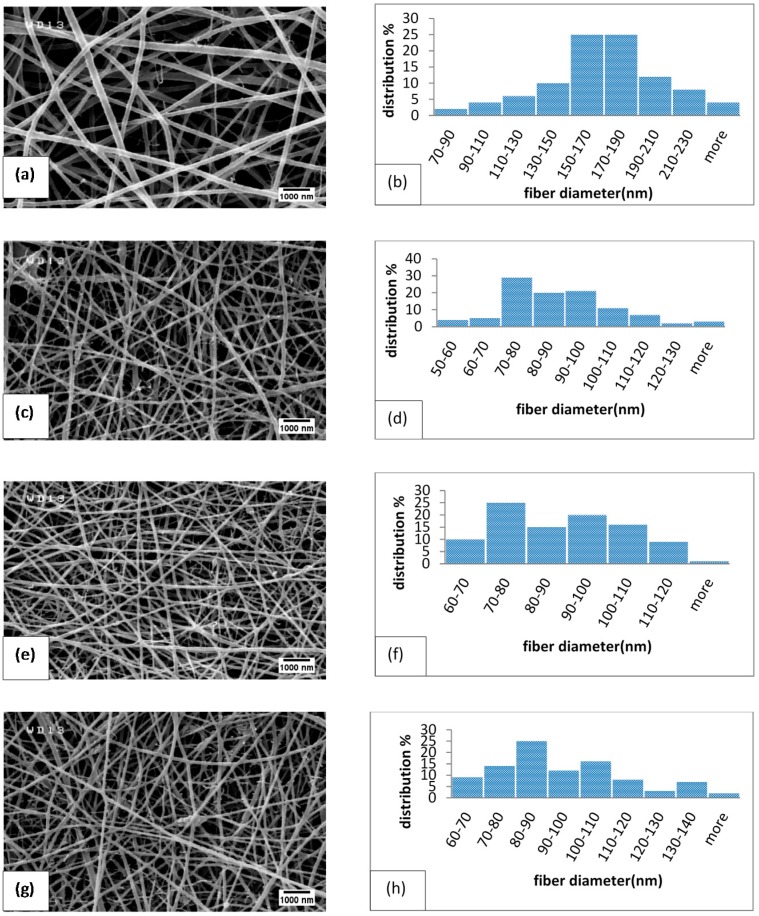
Effect of medical grade nanodiamonds (MND) on the morphology and size distribution of electrospun fibers: (**a**) chitosan/bacterial cellulose (CS/BC) without MND; (**c**) contain 1%; (**e**) 2% and (**g**) 3% MND particles, respectively. (**b**), (**d**), (**f**) and (**h**) show the fiber diameter distribution diagrams of each specimen.

**Figure 2:**
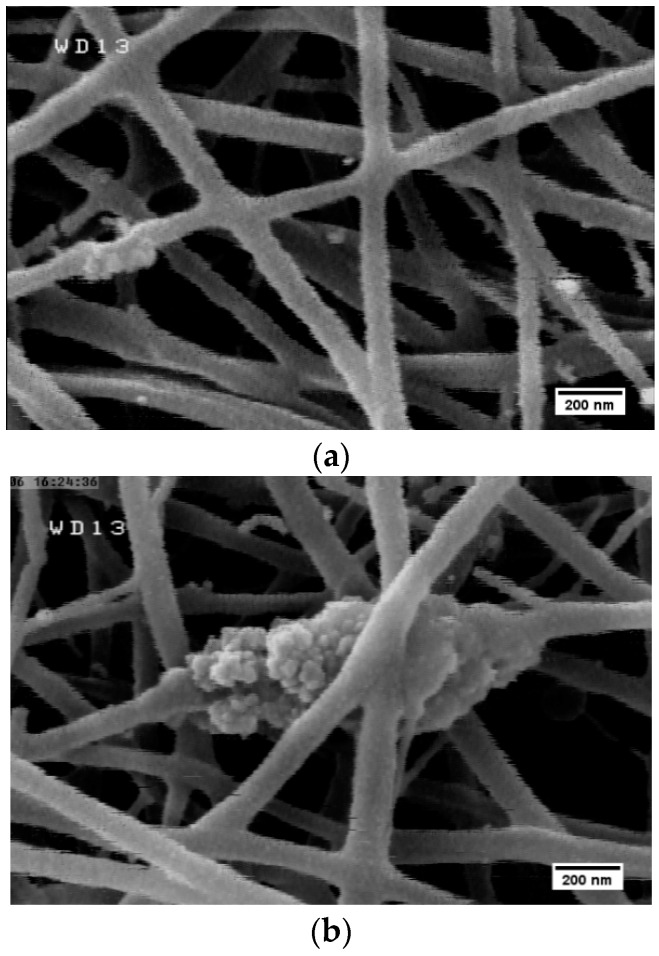
Formation of large nanoparticle clusters upon electrospinning. The concentration of MND (%) is (**a**) 2 and (**b**) 3.

Figure 1 shows representative SEM micrographs of electrospun fibers containing different amounts of MND. The size distribution of the fibers was determined by image analysis and is shown in Figure 1 and Table 1. Randomly oriented fibers with diameters ranging from 80 to 300 nm were obtained by electrospinning the polysaccharide suspension (Figure 1a,b). Some mini-jets were also visible, which could be due to the high viscosity of the suspension, i.e., the electrical field could not overcome surface tension forces [37]. Introduction of diamond nanoparticles reduced the fiber diameters and yielded more uniform fibers with narrower size distribution (Table 1). It is possible that the effect of MND on the fiber diameters is related to the enhanced viscosity and conductivity of the suspension from which the fibers are drawn. At a given electric field strength, the higher liquid viscosity typically results in a weaker stretching of liquid jet while higher conductivity changes the shape of meniscus [38]. The balance between the effect of nanoparticles on the viscosity and conductivity determines the size of the fibers that leads to the formation of coarser fibers [39]. As it will be shown in the next section, no strong chemical interactions between MND and polymer occurs, but agglomeration of the nanoparticles are favorable, particularly at high concentrations. For instance, Figure 2 shows the formation of MND clusters in electrospun fibers. Rakha et al. [40] related particles’ agglomeration to their surface functional groups. As the effective size of ultrafine MND increased, their effect on the viscosity decreased, so that finer fibers were attained [41].

### 2.2. Interactions between Nanoparticles and Polymer

**Figure 3:**
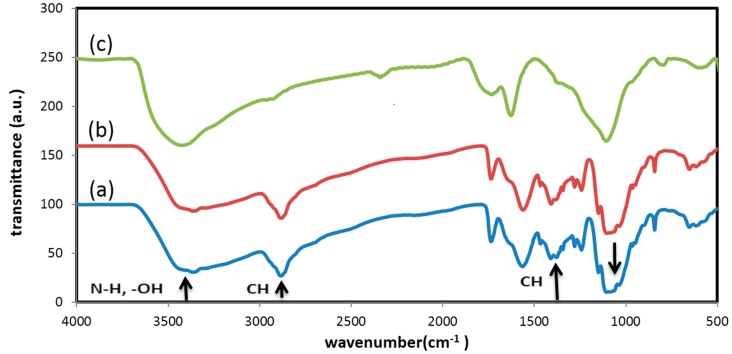
Fourier transform infrared (FT-IR) spectrum of (**a**) CS/BC polymer; (**b**) the nanocomposite fiber containing 3% MND; and (**c**) pristine MND.

In order to figure out the possible interactions between CS, BC, and MND particles, FT-IR spectroscopy was employed. Figure 3 shows FT-IR spectrum of the polymer blend, pristine MND particles, and electrospun fibers containing 3 wt % MND. The –OH stretching vibration of CS occurs at 3000–3500 cm^−1^ [25,42]. The peak around 2965 cm^−1^ is assigned to aliphatic C–H stretching vibration [31]. The N–H peak is for CS which overlaps the wide absorption peak of –OH group in range of 3000–3500 cm^−1^. The peaks at 1343 cm^−1^ and 1467 cm^−1^ are attributed to symmetric and bending vibration of C-H group, respectively. Another peak at 1050 cm^−1^ is due to the stretching vibration of C–O–C group. As to the PEO, triplet peaks of the C–O–C stretching vibrations appear at 1148, 1101, and 1062 cm^−1^. An absorption band at 2885 cm^−1^ is attributed to CH_2_ stretching vibration in PEO [43,44]. The peak of the glucose carbonyl of cellulose is detected at 1643 cm^−1^. The peak at around 1045 cm^−1^ shows the C–O–C stretching vibration. Overall, the results are in a good agreement with previous studies on CS and BC composite films and firmly verify the presence of many intermolecular hydrogen and ionic bonds, as well as a few covalent bonds [24,25,42]. The absorption peaks of MND are seen at 3426 cm^−1^, 1626 cm^−1^, and 1107 cm^−1^ which are attributed to stretching vibration of –OH, –C=O, and the overlap of C–O groups with nitrogen groups, respectively. The peak at 1735 cm^−1^ indicates stretching vibration of functional groups of C=O and –COOH and the peak at 2341 cm^−1^ is related to the absorption of CO_2_ [45,46,47]. The FT-IR results determined there was no formation of new peaks as a result of blending the polymers with MND; only a slight red shift in characteristics peaks were observed that could be due to hydrogen bonding [48].

### 2.3. Effect of Nanodiamonds on the Hydrophilicity of Mats

**Figure 4:**
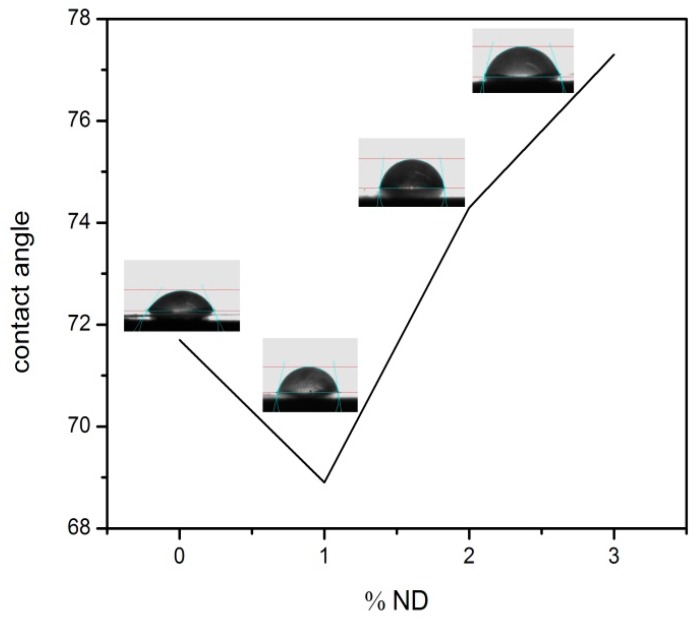
Effect of diamond particles on the hydrophilicity of electrospun CS/BC mats.

Figure 4 shows changes in the water contact angle of electrospun fibers with the MND concentration. The hydrophilicity at first decreased with the introduction of 1 wt % MND particles, but increased at the higher concentrations. The MND particles with many surface functional groups are hydrophilic but, at the high concentrations, the addition of these nanoparticles increases the hydrophobicity of the mats. It is suggested that changes in the contact angles were more likely due to the morphology/size variations of the fibers. It is known that the surface energy and surface geometric features influence the contact angles of fibrous mats [49,50]. Smaller fibers with agglomerated nanoparticles altered the hydrophilicity due to the roughness issue and surface features [51].

### 2.4. Mechanical Properties of Electrospun Mats

**Figure 5:**
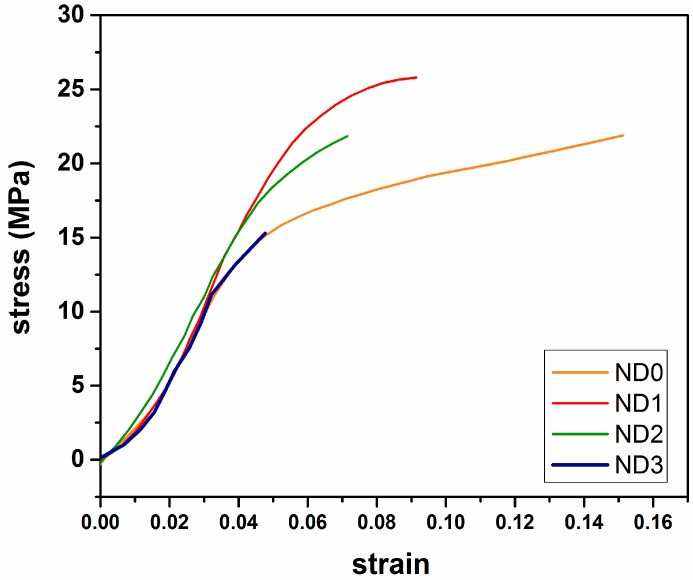
Stress-strain curves of electrospun mats containing different amounts of diamond nanoparticles.

Typical stress-strain curves of the electrospun mats are shown in Figure 5. The effect of MND on the mechanical properties are summarized in Table 2. It was found that the addition of 1 wt % MND particles significantly increased the elastic modulus and yield strength of the fibers along with ductility loss. Tjong et al. [52] related the mechanism of strength enhancement to the effect of nanoparticles on restricted movement of polymer chains. It was noted that the mechanical properties of the mats were close to that of natural skin, as reported in [27]. Meanwhile, the mechanical durability was degraded at high MND concentrations due to severe agglomeration of the nanoparticles. Herein, it should be noted that no chemical interactions occurred between the particles and the polymer matrix, so mechanical interlocking of the polymer chain by the nanoparticles could be the main mechanism of the enhanced durability.

### 2.5. Permeability

**Figure 6:**
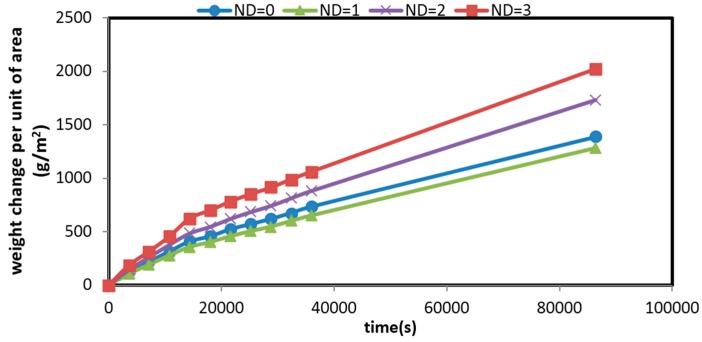
Weight change per unit of area of the mats versus time for CS/BC mats containing different amounts of MND.

The water vapor permeability of films was determined by a gravimetric method at room temperature. It is noteworthy that permeability of wound dressings is an important factor in the skin repairing process in order to create the appropriate moisture in wound area and stop over-drying [17]. Figure 6 shows weight change of the mats over time per unit area. The slope of the lines yields the average value of permeability (Table 2). It is seen that the permeability of the electrospun mats decreases with increasing the ND concentration. It is known that the permeability is related to the size and structure of pores in the fibrous mats [53]. As shown in Figure 1, the membranes containing MND exhibited finer fibers with a more closed-packed structure; hence, the permeability was reduced.

### 2.6. Cell Viability Assessment

**Figure 7:**
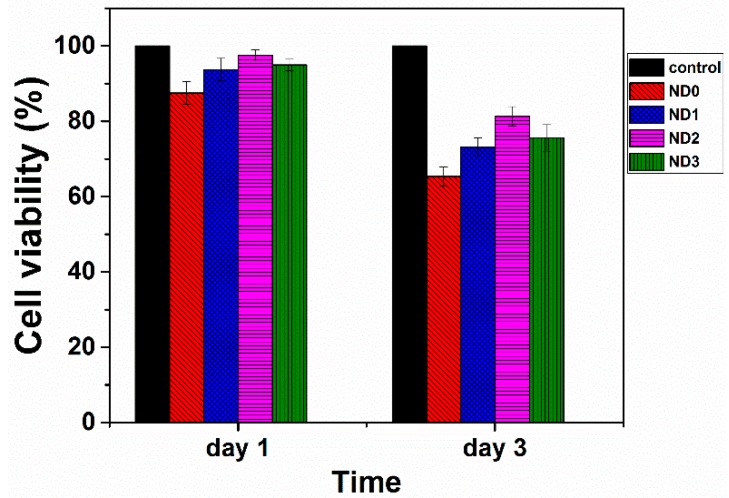
Cell viability of CS/BC mats dependent on the MND content at two incubated times.

Many studies have shown that diamond particles are cytocompatiable [54,55,56]. To be certain that embedding of these particles in the fibrous structure of CS/BC prepared by electrospinning does not degrade biocompatibility, MTT assay was performed. Figure 7 shows the cell viability of electrospun mats containing different amounts of MND after 24 and 72 h incubation. The viability varied in the range of 75%–90% of the control sample. As compared with previous studies [8], the prepared mats exhibited better cytocompatibility to L929 cells. The cell viability decreased when increasing the amount of MND beyond 2 wt %. This observation might be related to the agglomeration of the nanoparticles as well as refined fibrous structure with different pore sizes.

## 3. Experimental Procedure

### 3.1. Materials

Chitosan, with an average molecular weight of 90–150 kDa and degree of deacetylation of 75%–85%, was supplied by Sigma-Aldrich Co. (St. Louis, MO, USA). Bacterial cellulose nanofibers were purchased from Nano Novin Polymer Co., Sari, Iran. Medical grade diamond nanoparticles with an average particle size of 2–6 nm (Grade PL-D-G, purity > 87%) were supplied by PlasmaChem GmbH (Berlin, Germany). Acetic acid (100%) was obtained from Merck Co., Darmstadt, Germany. Polyethylene oxide (PEO), with a molecular weight of 900 kDa, was provided by Sigma-Aldrich Co. (St. Louis, MO, USA).

### 3.2. Preparation of Fibrous Mats

To prepare polymeric solutions for electrospinning, an aqueous solution of chitosan in acetic acid (3 wt %) was prepared. The solution was blended with a bacterial cellulose gel (5 wt %) dissolved in acetic acid (90%). A polyethylene oxide solution in deionized water (5 wt %) was separately prepared and added to the CS/BC suspension. The final composition of the suspension was 45:45:10 CS:BC:PEO. The aim of PEO addition was to reduce the viscosity of the gel and facilitate electrospinning [57].

MND-modified fibrous mats were prepared by electrospinning the polymeric suspension mixed with MND in a concentration of 1, 2, and 3 wt % of the polymer. The diamond nanoparticles were dispersed in deionized water by employing an ultrasonic bath (Wise Clean, Model: WUC-D10h using power of 690 W) for one hour. The dispersion was added to the polymeric suspension and sonicated for one hour.

A single jet electrospinning apparatus (Model HVPS-35/500, ANSTCO, Tehran, Iran) was employed to prepare fibrous mats. The suspension was fed into a horizontally aligned syringe with a needle orifice of 0.55 mm in inner diameter. The distance between syringe and collector was fixed at 10 cm and the applied voltage was 20–22 kV. The electrospinning feeding rate was 0.3 mL/h and the suspensions were spun toward a rotating drum (1000 rpm). The randomly oriented nanofibers were collected on an aluminum foil.

### 3.3. Materials Characterization

The size and morphology of the fibers were studied by field-emission scanning electron microscopy (FE-SEM; Hitachi Ltd., Tokyo, Japan, F-System 4160) at an acceleration voltage of 20 kV. Gold coating was applied before imaging. Fourier transform infrared (FT-IR) analysis was performed by a Spectrum 400 Perkin Elmer (Waltham, MA, USA) in the range of 450–4000 cm^−1^ with a resolution of 1 cm^−1^. A universal tensile testing machine (Instron 5566, Norwood, MA, USA) was employed to evaluate the mechanical properties i.e. ultimate Tensile Strength (UTS) and elongation to break, based on ASTM Standard D638. The examined mats had a thickness of 30 ± 2 µm and a gauge length of 20 mm. The crosshead speed was 5.0 mm/min. Each test was repeated three times and the average values were recorded.

To determine the hydrophilicity of the membranes, water contact measurement was performed by employing an OCA 15 plus video-based optical contact angle meter (Data Physics Instruments GmbH, Filderstadt, Germany). Water vapor permeability (WVP) was gravimetrically determined according to ASTM E96 desiccant method at room temperature. Films with an area of 3.5 cm^2^ were prepared and overlaid on cups filled with silica gel. The cups were placed in a desiccator containing saturated salt solution of ammonium nitrate with relative moisture 65%. At several time periods (1–24 h), the weight change was measured. Water vapor transmission (WVT) and permeability (WVP) were determined by [23]:

(1) WVT=Gt×A (gsec.m2)

(2) WVP=WVTΔP×T=WVTS(R1−R2)×T (gPa.sec.m)

where *G* is the weight gain of the cup at time *t*, A the permeation area, ΔP the vapor pressure difference across the mat—calculated based on the vapor saturation pressure (*S*) and the relative humidity inside (100%) and outside the cup (49%)—and *T* the average thickness of the mats.

### 3.4. Cell Viability

Cell viability was evaluated using the standard 3-(4,5-dimethylthiazol-2-yl)-2,5-diphenyl tetrazolium bromide (MTT) assay protocol. The assay is based on the conversion of MTT into formazan crystals by living cells, which determines mitochondrial activity. Briefly, 5 × 10^5^ mouse skin fibroblast cells (L929) (National Cell Bank, Iran Pasture Institute) were seeded onto the specimens with a 6-well plate and incubated at 37 °C in 5% CO_2_ for 24 and 48 h. After each interval, 200 mL of MTT solution (Sigma, St. Louis, MO, USA, 5 mg/mL) in 1× Dulbecco’s phosphate-buffered saline (PBS; Sigma, St. Louis, MO, USA) was added to each well and the cells were incubated for another 4 h. Upon removal of the MTT solution, the formed formazan crystals were solubilized with isopropanol for 15 min. Absorbance was read at the wavelength of 570 nm. The data were reported separately for each well by an ELISA reader (BioTek Microplate Reader, BioTek Company, Winooski, VT, USA). An average of triplicate wells was calculated, and the standard deviation for each sample was calculated based on Student’s *t*-test (*p* < 0.05). To observe the morphology of the adherent cells, the films were washed by PBS three times and then immersed in 3% glutaraldehyde PBS solution for 30 min for cell fixing. The films were dehydrated in ascending series of ethanol aqueous solutions (50 to 100 percent) at room temperature. The specimens were kept overnight in a desiccator to remove any moisture. The growth of the cells was observed after 24 h incubation.

## 4. Conclusions

Chitosan/bacterial cellulose nanofibrous membranes modified with diamond nanoparticles were fabricated by electrospinning. It was shown that uniform mats with an average fiber diameter of about 130 nm could be prepared at the spinning distance of 120 mm, applied voltage of 20 kV, and CS/BC ratio of 1:1. The effect of MND particles on the electrospinning of CS/BC blends was also studied. It was shown that MND particles reduced the average fiber diameter. The mechanical properties under tensile loading, the hydrophilicity, and water vapor permeability of the nanocomposites were also examined. An enhancement in the mechanical strength with a decrease in strain to failure was measured due to the addition of MND to the CS/BC nanofibers. Introduction of MND particles was accompanied with a gradual decrease in the hydrophilicity of the nanofibrous membranes as well. The water vapor permeability of the CS/BC nanofibers was also decreased. The cell viability of fibrous membrane after a 1-day incubation was >90% as compared with the control. We concluded that the fibrous mat containing 1% MND could be a promising candidate for wound dress and tissue engineering applications. Meanwhile, in vivo studies and pre-clinical investigations are required to explore the suitability of developed mats for biomedical applications.

## Figures and Tables

**Table 1:** Effect of medical grade nanodiamonds (MND) particles on size and size distribution of fibers.

Concentration	Average Fiber Diameter (nm)	Size Range (nm)
0	173 ± 44	73–308
1	88 ± 18	57–160
2	89 ± 14	61–128
3	95 ± 22	60–157

**Table 2:** Effect of MND on the mechanical properties and permeability of electrospun mats.

Concentration	Elastic Modulus (MPa)	Yield Strength (MPa)	Strain to Failure (%)	Permeability (µg·Pa^−1^·s^−1^·m^−1^)
0	353	21.7	15.4	423
1	458	25.3	9.9	345
2	393	20.2	7.9	342
3	405	15.9	4.8	359

## References

[1] Zahedi P., Rezaeian I., Ranaei-Siadat S.O., Jafari S.H., Supaphol P. (2010). A review on wound dressings with an emphasis on electrospun nanofibrous polymeric bandages. Polym. Adv. Technol..

[2] Kokabi M., Sirousazar M., Hassan Z.M. (2007). Pva-clay nanocomposite hydrogels for wound dressing. Eur. Polym. J..

[3] Jiang T., Carbone E.J., Lo K.W.-H., Laurencin C.T. (2015). Electrospinning of polymer nanofibers for tissue regeneration. Prog. Polym. Sci..

[4] Sun B., Jiang X.-J., Zhang S., Zhang J.-C., Li Y.-F., You Q.-Z., Long Y.-Z. (2015). Electrospun anisotropic architectures and porous structures for tissue engineering. J. Mater. Chem. B.

[5] Patrulea V., Ostafe V., Borchard G., Jordan O. (2015). Chitosan as a starting material for wound healing applications. Eur. J. Pharm. Biopharm..

[6] Azuma K., Izumi R., Osaki T., Ifuku S., Morimoto M., Saimoto H., Minami S., Okamoto Y. (2015). Chitin, chitosan, and its derivatives for wound healing: Old and new materials. J. Funct. Biomater..

[7] Mahmoudi N., Ostadhossein F., Simchi A. (2016). Physicochemical and antibacterial properties of chitosan-polyvinylpyrrolidone films containing self-organized graphene oxide nanolayers. J. Appl. Polym. Sci..

[8] Ostadhossein F., Mahmoudi N., Morales-Cid G., Tamjid E., Navas-Martos F.J., Soriano-Cuadrado B., Paniza J.M.L., Simchi A. (2015). Development of chitosan/bacterial cellulose composite films containing nanodiamonds as a potential flexible platform for wound dressing. Materials.

[9] Zhu W., Li W., He Y., Duan T. (2015). In-situ biopreparation of biocompatible bacterial cellulose/graphene oxide composites pellets. Appl. Surf. Sci..

[10] Pina S., Oliveira J.M., Reis R.L. (2015). Natural-based nanocomposites for bone tissue engineering and regenerative medicine: A review. Adv. Mater..

[11] Koosha M., Mirzadeh H. (2015). Electrospinning, mechanical properties, and cell behavior study of chitosan/PVA nanofibers. J. Biomed. Mater. Res. A.

[12] Jayakumar R., Prabaharan M., Kumar P.S., Nair S., Tamura H. (2011). Biomaterials based on chitin and chitosan in wound dressing applications. Biotechnol. Adv..

[13] Ibrahim H., El-Zairy E. (2015). Chitosan as a Biomaterial—Structure, Properties, and Electrospun Nanofibers. Intech.

[14] Jia Y., Huang G., Dong F., Liu Q., Nie W. (2015). Preparation and characterization of electrospun poly (ε-caprolactone)/poly (vinyl pyrrolidone) nanofiber composites containing silver particles. Polym. Compos..

[15] Haider S., Al-Masry W., Al-Zeghayer Y., Al-Hoshan M., Ali F.A. (2016). Fabrication chitosan nano fibers membrane via electrospinning. Une.

[16] Millon L.E., Guhados G., Wan W. (2008). Anisotropic polyvinyl alcohol—Bacterial cellulose nanocomposite for biomedical applications. J. Biomed. Mater. Res. B Appl. Biomater..

[17] Czaja W., Krystynowicz A., Bielecki S., Brown R.M. (2006). Microbial cellulose—The natural power to heal wounds. Biomaterials.

[18] Millon L., Wan W. (2006). The polyvinyl alcohol–bacterial cellulose system as a new nanocomposite for biomedical applications. J. Biomed. Mater. Res. B Appl. Biomater..

[19] Helenius G., Bäckdahl H., Bodin A., Nannmark U., Gatenholm P., Risberg B. (2006). In vivo biocompatibility of bacterial cellulose. J. Biomed. Mater. Res. A.

[20] Fu L., Zhang J., Yang G. (2013). Present status and applications of bacterial cellulose-based materials for skin tissue repair. Carbohydr. Polym..

[21] Jebel F.S., Almasi H. (2016). Morphological, physical, antimicrobial and release properties of ZnO nanoparticles-loaded bacterial cellulose films. Carbohydr. Polym..

[22] Römling U., Galperin M.Y. (2015). Bacterial cellulose biosynthesis: Diversity of operons, subunits, products and functions. Trends Microbiol..

[23] Fernandes S.C., Oliveira L., Freire C.S., Silvestre A.J., Neto C.P., Gandini A., Desbriéres J. (2009). Novel transparent nanocomposite films based on chitosan and bacterial cellulose. Green Chem..

[24] Phisalaphong M., Jatupaiboon N. (2008). Biosynthesis and characterization of bacteria cellulose–chitosan film. Carbohydr. Polym..

[25] Lin W.-C., Lien C.-C., Yeh H.-J., Yu C.-M., Hsu S.-H. (2013). Bacterial cellulose and bacterial cellulose–chitosan membranes for wound dressing applications. Carbohydr. Polym..

[26] Zhang P., Chen L., Zhang Q., Hong F.F. (2016). Using in situ dynamic cultures to rapidly biofabricate fabric-reinforced composites of chitosan/bacterial nanocellulose for antibacterial wound dressings. Front. Microbiol..

[27] Azarniya A., Eslahi N., Mahmoudi N., Simchi A. (2016). Effect of graphene oxide nanosheets on the physico-mechanical properties of chitosan/bacterial cellulose nanofibrous composites. Compos. A Appl. Sci. Manuf..

[28] Ahmed F.E., Lalia B.S., Hashaikeh R. (2015). A review on electrospinning for membrane fabrication: Challenges and applications. Desalination.

[29] HPS A.K., Saurabh C.K., Adnan A., Fazita M.N., Syakir M., Davoudpour Y., Rafatullah M., Abdullah C., Haafiz M., Dungani R. (2016). A review on chitosan-cellulose blends and nanocellulose reinforced chitosan biocomposites: Properties and their applications. Carbohydr. Polym..

[30] Khalil H.A., Davoudpour Y., Bhat A., Rosamah E., Tahir P.M. (2015). Electrospun cellulose composite nanofibers. Handbook of Polymer Nanocomposites. Processing, Performance and Application.

[31] Liu Y., Zhou J., Tang J., Tang W. (2015). Three-dimensional, chemically bonded polypyrrole/bacterial cellulose/graphene composites for high-performance supercapacitors. Chem. Mater..

[32] Kalashnikova I., Das S., Seal S. (2015). Nanomaterials for wound healing: Scope and advancement. Nanomedicine.

[33] Hsiao S.-T., Ma C.-C.M., Tien H.-W., Liao W.-H., Wang Y.-S., Li S.-M., Chuang W.-P. (2015). Preparation and characterization of silver nanoparticle-reduced graphene oxide decorated electrospun polyurethane fiber composites with an improved electrical property. Compos. Sci. Technol..

[34] Schrand A.M., Dai L., Schlager J.J., Hussain S.M., Osawa E. (2007). Differential biocompatibility of carbon nanotubes and nanodiamonds. Diam. Relat. Mater..

[35] Zhu Y., Li J., Li W., Zhang Y., Yang X., Chen N., Sun Y., Zhao Y., Fan C., Huang Q. (2012). The biocompatibility of nanodiamonds and their application in drug delivery systems. Theranostics.

[36] Kaur R., Badea I. (2013). Nanodiamonds as novel nanomaterials for biomedical applications: Drug delivery and imaging systems. Int. J. Nanomed..

[37] Hsu C.M., Shivkumar S. (2004). *N*,*N*-dimethylformamide additions to the solution for the electrospinning of poly (ε-caprolactone) nanofibers. Macromol. Mater. Eng..

[38] Li X., Bian F., Lin J., Zeng Y. (2016). Effect of electric field on the morphology and mechanical properties of electrospun fibers. RSC Adv..

[39] Naseri N., Mathew A.P., Girandon L., Fröhlich M., Oksman K. (2015). Porous electrospun nanocomposite mats based on chitosan–cellulose nanocrystals for wound dressing: Effect of surface characteristics of nanocrystals. Cellulose.

[40] Rakha S.A., Raza R., Munir A. (2013). Reinforcement effect of nanodiamond on properties of epoxy matrix. Polym. Compos..

[41] Kartikowati C.W., Suhendi A., Zulhijah R., Ogi T., Iwaki T., Okuyama K. (2015). Preparation and evaluation of magnetic nanocomposite fibers containing α″-Fe_16_N_2_ and α-Fe nanoparticles in polyvinylpyrrolidone via magneto-electrospinning. Nanotechnology.

[42] Ul-Islam M., Shah N., Ha J.H., Park J.K. (2011). Effect of chitosan penetration on physico-chemical and mechanical properties of bacterial cellulose. Korean J. Chem. Eng..

[43] Mishra R., Rao K.J. (1999). On the formation of poly (ethyleneoxide)-poly (vinylalcohol) blends. Eur. Polym. J..

[44] Duan B., Dong C., Yuan X., Yao K. (2004). Electrospinning of chitosan solutions in acetic acid with poly (ethylene oxide). J. Biomater. Sci. Polym. Ed..

[45] Osswald S., Yushin G., Mochalin V., Kucheyev S.O., Gogotsi Y. (2006). Control of sp^2^/sp^3^ carbon ratio and surface chemistry of nanodiamond powders by selective oxidation in air. J. Am. Chem. Soc..

[46] Morimune S., Kotera M., Nishino T., Goto K., Hata K. (2011). Poly (vinyl alcohol) nanocomposites with nanodiamond. Macromolecules.

[47] Zou Q., Li Y., Zou L., Wang M. (2009). Characterization of structures and surface states of the nanodiamond synthesized by detonation. Mater. Charact..

[48] Pan H., Xu D., Liu Q., Ren H.Q., Zhou M. (2014). Preparation and Characterization of Corn Starch-Nanodiamond Composite Films. Applied Mechanics and Materials.

[49] Wang X., Yu J., Sun G., Ding B. (2015). Electrospun nanofibrous materials: A versatile medium for effective oil/water separation. Mater. Today.

[50] Wang Z., Macosko C.W., Bates F.S. (2016). Fluorine enriched melt blown fibers from polymer blends of poly (butylene terephthalate) and a fluorinated multiblock copolyester. ACS Appl. Mater. Interfaces.

[51] Ma M., Mao Y., Gupta M., Gleason K.K., Rutledge G.C. (2005). Superhydrophobic fabrics produced by electrospinning and chemical vapor deposition. Macromolecules.

[52] Tjong S. (2006). Structural and mechanical properties of polymer nanocomposites. Mater. Sci. Eng. R Rep..

[53] Cui W., Zhou Y., Chang J. (2016). Electrospun nanofibrous materials for tissue engineering and drug delivery. Sci. Technol. Adv. Mater..

[54] Lee D.-K., Kim S.V., Limansubroto A.N., Yen A., Soundia A., Wang C.-Y., Shi W., Hong C., Tetradis S., Kim Y. (2015). Nanodiamond–gutta percha composite biomaterials for root canal therapy. ACS Nano.

[55] Hsiao W.W.-W., Hui Y.Y., Tsai P.-C., Chang H.-C. (2016). Fluorescent nanodiamond: A versatile tool for long-term cell tracking, super-resolution imaging, and nanoscale temperature sensing. Acc. Chem. Res..

[56] Taylor A.C., Vagaska B., Edgington R., Hébert C., Ferretti P., Bergonzo P., Jackman R.B. (2015). Biocompatibility of nanostructured boron doped diamond for the attachment and proliferation of human neural stem cells. J. Neural Eng..

[57] Rieger K.A., Birch N.P., Schiffman J.D. (2016). Electrospinning chitosan/poly (ethylene oxide) solutions with essential oils: Correlating solution rheology to nanofiber formation. Carbohydr. Polym..

